# Health-related quality of life of children and adolescents with mental disorders

**DOI:** 10.1186/1477-7525-11-129

**Published:** 2013-07-31

**Authors:** Katharina Weitkamp, Judith K Daniels, Georg Romer, Silke Wiegand-Grefe

**Affiliations:** 1 Department of Child and Adolescent Psychiatry, Psychotherapy and Psychosomatics, University Medical Centre Hamburg-Eppendorf, Hamburg, Germany; 2 Department of Psychiatry, Universitätsmedizin Charité, Berlin, Germany; 3 Department of Child and Adolescent Psychiatry, Psychosomatics and Psychotherapy, University Hospital Münster, Münster, Germany

**Keywords:** Quality of life, Internalising disorders, Externalising disorders, Child, Adolescent

## Abstract

**Background:**

The aim was to assess the association of internalising and externalising pathology with the child’s health-related quality of life (QoL), and to determine which child and environmental characteristics beyond pathology were related to poor QoL.

**Methods:**

Data was obtained for 120 children and adolescents (aged 6 to 18) commencing outpatient psychotherapy treatment. Parents and children (aged 11 years and older) filled out questionnaires. QoL was measured with the KIDSCREEN-27.

**Results:**

QoL was more strongly associated with internalising than externalising pathology according to both self- and parent report. Multiple regression analyses showed that beyond internalising and externalising pathology, gender, age, family functioning, functional impairment, and prior mental health treatment were associated with individual QoL scales.

**Conclusions:**

The data underscored the relationship between mental pathology and impaired QoL even if potential item overlap was controlled for. This stresses the importance of extending therapy goals and outcome measures from mere pathology to measures of QoL in psychotherapy research particularly for patients with internalising pathology.

## Introduction

Research on health-related quality of life (QoL) in children and adolescents with psychiatric disorders is still in its early stages. Though in evidence-based assessment it has been suggested to supplement the measure of pathology with the assessment of QoL and functioning in child psychotherapy research [1]. This seems warranted, since suffering from a psychiatric disorder during childhood and adolescence has a considerable impact on the child’s subjective satisfaction with his or her day-to-day activities and social well-being [2,3]. QoL in children with mental health problems is not only considerably poorer than in healthy children, but QoL seems to be more severely impaired even when compared with children suffering from a chronic somatic illness [4,5].

It seems critical to search for influencing factors beyond the specific association with pathology so that therapists might respond in a more targeted way. In a first study on factors influencing QoL in children with a psychiatric disorder, Bastiaansen and his colleagues [6] noted that the impact of psychopathology was larger in girls than in boys, possibly because children with externalising behaviour problems (predominantly males) may not experience their symptoms as problematic. Secondly, the impact of psychopathology increased with age. However, a mediation by chronicity of the disorder was not tested. Furthermore, poor global report of QoL co-occurred with poor social support, poor family functioning, and stressful life events.

There is conflicting evidence on whether different mental disorders have a specific impact on QoL. Schubert and colleagues [7] found no differences between diagnostic groups of children suffering from either emotional, somatoform, or externalising disorders. Contrary to this, two further studies reported lower levels of emotional functioning, more pain, and increased discomfort in children with depressive symptoms [4,5]. In contrast, school functioning and social functioning were more likely affected in children with attention-deficit and disruptive behaviour disorders [4,5].

A criticism of previous QoL research is the failure to control for item overlap between QoL and mental health assessment [8]. To our knowledge, only two studies controlled for item overlap [5,9], reporting similar relationships between mental disorders and QoL.

The aim of the current study was to assess the differential impact of internalising and externalising pathology on the child’s QoL and to follow up on Bastiaansen’s [6] finding that diminished global QoL was associated with child factors beyond pathology. The above-mentioned study had several limitations concerning the informant type (relying on a mixture of sources for the predictor variables like parent, teacher, clinician, and child report) and the undifferentiated consideration of QoL with a global QoL score.

The following hypotheses were tested in the current study: (a) both, internalising and externalising pathology are significantly related to the QoL dimensions; (b) family functioning, chronicity of pathology indicated by prior mental health treatment, level of impairment, age and/or gender have an independent relationship with QoL, beyond the influence of externalising and internalising pathology, and (c) the association between QoL and pathology remains after controlling for item overlap between the constructs.

## Methods

### Procedure

Data collection was carried out as part of an effectiveness trial for child and adolescent psychotherapy in Germany. At the commencement of the outpatient therapy, patients (11 years and older) and both parents (if available) were asked to participate by the therapist. Between September 2007 and June 2010, 120 of the approached 272 families with a child between 6 to 18 years agreed to participate and gave their written informed consent (44.1%), returned their questionnaires, and hence compose the sample of the current study. A further 152 families refused to participate (55.9%). For these patients we attained anonymous basic data on age, gender, and diagnostic status from the therapists. Comparison of the participants and non-participants yielded no significant differences in terms of age, gender, and comorbidity status, and frequency of disorders other than affective disorders. Affective disorders were significantly more prevalent in the group of the participants (31% vs. 19.7%). The study was approved by the ethics committee of the Medical Board Hamburg.

### Participants

Reporting on a total of 120 patients, 73 children/adolescents (11 years and older) and 103 parents filled out questionnaires. The sample was on average *M*_age_ = 12.55 years old (range: 6–18 years), 63.5% were female (n = 77), 51% came from divorced families. All patients had at least one diagnosed mental disorder, 67.3% reported comorbidities. The therapists diagnosed 45.1% with an anxiety disorder, 31.0% with an affective disorder, 25.7% with a PTSD, 15.9% with a disruptive disorder, and 33.6% with other disorders (mainly eating disorders, enuresis, encopresis, or sleeping disorders).

### Measures

The assessment instruments presented in the current study was part of a broader assessment battery compiled for a psychotherapy effectiveness trial.

Health-related quality of life was assessed with the German KIDSCREEN-27 [2]. This instrument consists of 27 items and was developed to measure five dimensions: *physical well-being, psychological well-being, autonomy and parent relation, social support and peers*, as well as *school environment*. Parallel parent and child self-rating versions are available. Each item is scored on a 5-point Likert scale ranging from 1 = ‘not at all/never’ to 5 ‘extremely/always’. The items are summed up for the specific subscales with high values representing high levels of QoL and may be converted into T-values (T > 45 are considered above average). Reliability has been shown to be good (Cronbach’s alpha: α = .78 to α = .83).

To assess the overall pathology, subjects were administered the Child Behavior Checklist for parents (CBCL; [10]) or Youth Self Report for children and adolescents (YSR; [11]), respectively. The CBCL/YSR consists of 118 items on specific emotional and behavioural problems in childhood and adolescence. An internalising and an externalising symptom score can be calculated from the corresponding syndrome scales. Each item stands for a specific problem behaviour and is rated on a 3-point scale from 0 = ‘not true’ to 2 = ‘very true or often true’. The reliability and validity of these widely used instruments have been examined in a number of studies [11,12].

Level of functional impairment was rated by the therapists on the Impairment-Score for Children and Adolescents (IS-CA; [13]). The IS-CA measures functional impairment on four dimensions: mental, somatic, social-communicative, and performance. Each item is rated on a five-point scale ranging from 0 = ‘not at all’ to 4 = ‘extremely’ and may be summed up to a total score. Higher scale scores (ranging from 0 to 24) stand for greater functional impairment (cut-off ≥ 7; [14]). Retest-reliability was high for the total score, *r*_tt_ = .84 [13].

Family functioning was assessed with the Familienboegen (FB; [15]). The FB is a self-report scale comprising 28 items which family members rate on a Likert-scale ranging from 0 = ‘completely true’ to 3 = ‘not true at all’. The seven subscales (*communication, role behaviour, task fulfilment, emotionality, control, values and norms,* and *affective establishment of relations*) may be summed into a total score and transformed into T-values. Higher scale scores reflect greater dysfunction (cut-off T ≥ 60; [15]). In the current study, reliability for the total score was satisfying with α = .79.

As an indicator of chronicity of pathology we assessed whether the youth had prior mental health treatment with an ad hoc formulated item asking if and when the patient had previously received treatment for a mental health condition. The item was dichotomous ‘yes/no’.

### Analyses

Data were processed with SPSS 18.0. Hierarchical linear regression analyses were calculated with the QoL scales as dependent variables and child characteristics as independent variables for the patient and the parent report and for each subscale separately. For the analyses of the parent report n = 103 cases were included, for the patient report n = 73 cases could be included. The independent variables were centred on the individual mean. The potential bias due to multicollinearity seemed negligible (1.078 < variance inflation factor < 1.478). Test power was sufficient (> 90%) (GPower; [16]). The independent variables were entered in two subsequent blocks into the regression model. First, internalising and externalising pathology were entered. In the second block, age, gender, functional impairment, perceived family functioning, and prior treatment of a mental health condition were entered. Results and effect sizes were evaluated based on established conventions [17]. To control for potential item overlap, we excluded one item on the ability to concentrate in the scale *school environment* and three items on depressed mood in the scale *psychological well-being.* For these two scales regression analyses were repeated and results compared with results for the raw scores of the complete scales. Raw scores instead of T-values were chosen for comparability reasons.

## Results

### Sample description

See Table 1 for descriptive statistics of the scales. Compared with norm data, patients reported reduced levels of QoL regarding *physical* and *psychological well-being* as well as satisfaction with *social support and peers*. The parents reported reduced levels of their child’s QoL for *physical* and *psychological well-being* as well as for the satisfaction with the *school environment.* Furthermore, patients and parents reported on average clinical levels of internalising pathology and elevated but not clinical levels of externalising pathology. Functional impairment as rated by the therapists was high and in the clinical range. Additionally, family functioning was reported to be in the normative range. About a third of the sample indicated having had prior mental health treatment.

**Table 1:** Means, standard deviations or proportions of QoL measures and predictor variables

	**Instrument**	**Mean/proportion**	**SD**
**Quality of Life (self-report)**	KIDSCREEN-27^a^		
Physical Well-being		40.81	8.86
Psychological Well-being		40.45	8.36
Autonomy & Parent Relations		46.29	7.66
Social Support & Peers		44.07	9.88
School Environment		45.16	7.89
**Quality of Life (parent report)**	KIDSCREEN-27^a^		
Physical Well-being		43.21	9.55
Psychological Well-being		38.15	8.37
Autonomy & Parent Relations		49.99	7.31
Social Support & Peers		46.86	10.31
School Environment		43.57	10.19
**Predictor variables**			
Internalising pathology	YSR/self-report^b^	62.87	12.22
	CBCL/parent report^b^	66.53	8.81
Externalising pathology	YSR/self-report^b^	54.90	7.78
	CBCL/parent report^b^	59.82	9.35
Patient age		12.55	3.65
Patient sex (female)		63.5%	
Level of functional impairment	IS-CA^c^	9.51	3.40
Family functioning	FB^b^	54.10	11.76
Prior mental health services	ad-hoc item	32.1%	

*Note*: a) cut-off T > 45 above average; b) T-scores, cut-off > 60 clinical/dysfunctional range; c) cut-off > 7 clinical range; YSR = Youth Self Report, CBCL = Child Behavior Checklist, IS-CA = Impairment-Score for Children and Adolescents; self-report n = 73, parent report n = 103.

### Patient report

The first step of the hierarchical regression analyses (see Table 2) tests whether high levels of internalising and externalising pathology were related to low levels of QoL. When entered simultaneously, mainly internalising pathology was related to low levels of QoL. For the children’s self-report, high levels of internalising pathology were significantly related to lower *psychological well-being* (β = −.69), as well as lower well-being in the realms of *social support and peers* (β = −.47) and *school environment* (β = −46). High levels of externalising pathology were significantly related only to lower well-being with *parent relations and autonomy* (β = −.32). *Physical well-being* was neither significantly associated with internalising nor with externalising pathology. Psychopathology explained a significant amount of variance of each QoL scale (between *R*^2^ = .13 for *autonomy and parent relations* and *R*^2^ = .61 for *psychological well-being).*

**Table 2:** Multiple linear regression analyses of factors associated with QoL – patient report

**Variables**	**QoL scales**														
	**Psychological well-being**			**Physical well-being**			**Autonomy & parent relations**			**Social support & peers**			**School environment**		
**Variable**	**β**	***SE***	***p***	**β**	***SE***	***p***	**β**	***SE***	***p***	**β**	***SE***	***p***	**β**	***SE***	***p***
Block 1 (psychopathology) *R*^2^	**.61**			**.22**			**.13**			**.17**			**.24**		
Internal. pathology	**-.69**	0.07	<.001	-.22	0.09	.117	.02	0.07	.893	**-.47**	0.12	.002	**-.46**	0.09	.004
External. pathology	-.00	0.12	.968	-.21	0.15	.103	**-.32**	0.12	.008	-.00	0.20	.975	-.04	0.15	.765
Block 2 (child factors) *R*^2^ change	.05			.16			**.36**			.15			.05		
Age	-.17	0.44	.091	**-.32**	0.61	.018	-.16	0.47	.186	.24	0.77	.076	-.12	0.58	.385
Gender	-.02	1.78	.828	.17	2.46	.193	**-.25**	1.88	.033	-.26	310	.053	-.08	2.24	.556
Functional impairment	-.13	0.26	.204	-.18	0.35	.205	-.02	0.28	.858	-.20	0.46	.184	.04	0.34	.806
Family functioning	.00	0.07	.991	-.12	0.09	.390	**-.56**	0.07	<.001	.08	0.12	.584	-.20	0.09	.188
Previous treatment	-.12	1.64	.203	.03	2.23	.837	-.10	1.75	.381	.06	2.86	634	.04	2.10	.771
Cumulative *R*^2^	**.66**			**.39**			**.48**			**.32**			**.30**		
Adjusted *R*^2^	**.60**^**a**^			**.29**^**b**^			**.40**^**c**^			**.21**^**d**^			**.17**^**e**^		

*Note*: *SE* = standard error of unstandardised B; standardised betas are presented for the full model; both significant and non-significant betas are presented; significant coefficients are printed in bold; dichotomous variables: gender (female = 0, male = 1), previous mental health treatment (no previous treatment = 0, previous treatment = 1).

^a^*F* = 11.767; *p* < .001.

^b^*F* = 3.877; *p* ≤ .002.

^c^*F* = 5.905; *p* < .001.

^d^*F* = 2.911; *p* ≤ .014.

^e^*F* = 2.393; *p* ≤ .038.

Few child factors explained some of the variance over and above the association between child psychopathology and QoL (see Table 2). Older age was associated with lower levels of *Physical well-being* (β = −.32). Male gender (β = −.25) and problematic family functioning (β = −.56) were associated with low well-being with *parent relations and autonomy.* The subscales *psychological well-being*, *social support and peers,* and *school environment* did not show a significant increment after the inclusion of child factors. Overall, between 17% (for *school environment)* and 60% variance (*psychological well-being)* could be explained by pathology and other child characteristics.

### Parent report

The first step of the hierarchical regression analyses tests whether high levels of internalising and externalising pathology were related to low levels of QoL for the parent report (see Table 3). Internalising and externalising pathology were significantly related to *psychological well-being* (internalising pathology: β = −.33) and *school environment* (externalising pathology: β = −.32), when entered simultaneously. The explained variance in the regression model was only significant for *psychological well-being* (*R*^2^ = .16) and QoL with *social support and peers* (*R*^2^ = .08)*.*

**Table 3:** Multiple linear regression analyses of factors associated with QoL – parent report

**Variables**	**QoL scales**														
	**Psychological well-being**			**Physical well-being**			**Autonomy & parent relations**			**Social support & peers**			**School environment**		
**Variable**	**β**	***SE***	***p***	**β**	***SE***	***p***	**β**	***SE***	***p***	**β**	***SE***	***p***	**β**	***SE***	***p***
Block 1 (psychopathology) *R*^2^	**.16**			.07			.05			**.08**			.07		
Internal. pathology	**-.33**	0.11	.006	-.14	0.11	.150	.02	0.11	.849	-.24	0.16	.064	.14	0.16	.270
External. pathology	-.08	0.10	.479	.05	0.10	.596	-.13	0.10	.294	.07	0.14	.566	**-.32**	0.15	.016
Block 2 (child factors) *R*^2^ change	**.15**			.43			.11			.10			**.18**		
Age	**-.32**	0.22	.002	**-.54**	0.22	<.001	.09	0.23	.436	-.17	0.32	.126	**-.30**	0.34	.010
Gender	-.12	1.64	.232	.06	1.62	.471	.04	1.65	.740	-.05	2.39	.683	-.22	2.36	.057
Functional impairment	-.19	0.25	.073	-.11	0.25	.227	-.11	0.25	.312	-.22	0.36	.054	**-.23**	0.37	.045
Family functioning	-.08	0.08	.455	-.07	0.08	.417	**-.33**	0.08	.006	-.11	0.11	.326	-.04	0.11	.763
Previous treatment	.04	1.77	.698	**-.20**	1.75	.031	.12	1.76	.323	-.02	2.54	.893	-.04	2.46	.760
Cumulative *R*^2^	**.30**			**.50**			.16			**.18**			**.25**		
Adjusted *R*^2^	**.24**^**a**^			**.45**^**b**^			.08^c^			**.11**^**d**^			**.18**^**e**^		

*Note*: *SE* = standard error of unstandardised B; standardised betas are presented for the full model; both significant and non-significant betas are presented; significant coefficients are printed in bold; dichotomous variables: gender (female = 0, male = 1), previous mental health treatment (no previous treatment = 0, previous treatment = 1).

^a^*F* = 4.799; *p* < .001.

^b^*F* = 10.937; *p* < .001.

^c^*F* = 2.082; *p* ≤ .056.

^d^*F* = 2.436; *p* ≤ .026.

^e^*F* = 3.271; *p* ≤ .005.

Few child factors explained some of the variance of the QoL scales for the parent report, over and above the association with child psychopathology (see second block of the regression model in Table 3). Older age (β = −.54) and previous mental health treatment (β = −.20) were associated with low levels of *physical well-being.* Problematic family functioning (β = −.33) was associated with low well-being with *parent relations and autonomy.* Additionally, older age (β = −.30) and higher functional impairment (β = −.23) were associated with lower well-being with the *school environment*. For QoL of *social support and peers* and *psychological well-being,* the inclusion of child characteristics yielded no significant increment beyond the variance explained by pathology. Overall, between 8% (for well-being with *autonomy and parent relations;* not significant, p ≤ .056) and 45% variance (for *physical well-being)* could be explained by pathology and other child characteristics as rated by their parents.

### Control for item overlap

The exclusion of overlapping items of the KIDSCREEN scales on *psychological well-being* and *school environment* did not change the regression models neither in significance nor magnitude compared with the raw data scales or the T-value scales for both self- and parent report (see Table 4 for regressions with raw scores and item overlap controlled scales of the self-report). For instance, prediction of self-reported *psychological well-being* was associated significantly (all *p* < .001) with internalising pathology in all three tested scale types: β_T-values_ = -.69; β_raw-values_ = −.71; β_overlap-control_ = −.72.

**Table 4:** Multiple linear regression analyses of factors associated with QoL for psychological well-being and school environment comparing raw scores and item overlap control – patient report

	***Psychological well-being***						***School environment***					
	**Raw scores**			**Item overlap control**			**Raw scores**			**Item overlap control**		
**Variable**	**β**	***SE***	***p***	**β**	***SE***	***p***	**β**	***SE***	***p***	**β**	***SE***	***p***
Block 1 (psychopathology) *R*^2^	**.61**			**.61**			**.23**			**.20**		
Internal. pathology	**-.71**	0.01	<.001	**-.72**	0.01	<.001	**-.50**	0.01	.001	**-.47**	0.01	.002
External. pathology	.01	0.01	.870	.05	0.01	.556	.03	0.02	.811	.03	0.02	.806
Block 2 (child factors) *R*^2^ change	.04			.03			.05			.05		
Age	-.14	0.03	.131	-.15	0.03	.109	.05	0.05	.734	.05	0.05	.694
Gender	-.03	0.17	.772	-.01	0.18	.897	-.15	0.23	.237	-.16	0.24	.213
Functional impairment	-.16	0.02	.097	-.10	0.02	.305	-.02	0.03	.917	-.01	0.04	.930
Family functioning	.08	0.01	.389	.07	0.01	.434	-.18	0.01	.207	-.19	0.01	.185
Previous treatment	-.06	0.15	.488	-.07	0.16	−413	.00	0.20	.979	.02	0.22	.888
Cumulative *R*^2^	**.65**			**.64**			**.28**			**.25**		
Adjusted *R*^2^	**.60**			**.59**			**.18**			**.15**		

## Discussion

The aim of the study was to assess the association of internalising and externalising pathology and the child’s QoL and to determine whether QoL was associated with child factors such as age, gender, functional impairment, poor family functioning or previous mental health treatment as an indicator of chronic psychiatric pathology.

As postulated in hypothesis (a), both, internalising and externalising pathology were associated with the child’s well-being and functioning. However, QoL showed a stronger association with internalising than externalising pathology for the self-report. Neither internalising nor externalising pathology was associated with every dimension of QoL.

Internalising pathology was related to diminished QoL in terms of psychological well-being, social support and peers as well as well-being with the school environment with moderate to large effect sizes. These findings are consistent with the Australian national survey results [5]. However, in contrast to our results, in a Dutch sample of children with anxiety and mood disorders QoL was mainly affected in terms of emotional functioning. Physical, social, and school functioning were not associated with overall pathology [4]. Likewise, no association was found between physical well-being and either internalising or externalising pathology in our sample of both patient and parent report.

Externalising pathology was associated with low levels of perceived quality of parent relations and autonomy. From the parents’ perspective, externalising problem behaviour was associated with low functioning at school. The latter finding mirrors the results of the Bastiaansen study [4]. But the findings contrast with the Australian study [5] where externalising pathology was not related to parent-rated peer contacts or school activities.

The current findings contradict the results of Schubert and colleagues [7] who stress that it is not so much the diagnosis but the overall impairment which diminishes QoL of children. In our study, particularly internalising pathology seemed to be related to impaired levels of well-being and functioning. Since Schubert and colleagues relied solely on parent reports, this fine-grained picture of differential impairment of QoL might not have been visible. Compared with the self-report, the parent reported pathology showed less association with the different aspects of the QoL. Another explanation for the stronger association of internalising pathology with QoL compared with externalising pathology might be an artefact due to the more pronounced internalising symptomatology of the sample. However, the distribution of the externalising scores covered a similar range compared with internalising pathology, showed slightly less variance for the self-report but not for the parent report. Thus, for the self-report the estimation of the association of QoL with externalising problems might be conservatively biased.

Hypothesis (b) was partly supported by the current data. Low family functioning and male gender were related to lower levels of the child’s well-being and functioning beyond the association with pathology. Consistent with Bastiaansen et al.’s findings [6], family and social network factors were associated with diminished QoL. In contrast to Bastiaansen et al. [6] who analysed QoL with a global score, this study investigated different facets of QoL separately with the subscales of the KIDSCREEN-27. Our data indicate a differential association of child and family characteristics with these facets. For instance, well-being with the school environment was particularly associated with internalising pathology. Problematic family functioning, e.g. dealing with conflicts or family rules, was associated with the child's experience of autonomy and relations to parents. The T-scores of the KIDSCREEN were already adjusted for age and gender, thus the influence of age and gender on the QoL ratings beyond the effect of externalising pathology was expectably low. Nevertheless, male gender was associated with more negative parent relations and less experience of autonomy as previously reported by Bastiaansen et al. [6]. The effect of age on the association of pathology and QoL reported by Bastiaansen et al. was replicated as follows. Both prior mental health treatment as a rough indicator of chronicity of mental pathology and age had an independent association with physical well-being in the parent report. This might indicate that in more chronic conditions of mental health issues the improvement of QoL could be worth considering in the therapeutic process independent of targeting pathology. However, because of the dichotomous and retrospective nature of the item on prior treatment, the influence might be underestimated and should be interpreted with caution.

Hypothesis (c) was supported by the current findings. Item overlap control did not change the regression models neither in significance nor magnitude compared with the raw data scales or the T-value scales for both self- and parent report. Item overlap did not change the power of the predictor variables in this sample. As previously shown by Sawyer et al. [18] and Dey and colleagues [9] for attention deficits, controlling for potential confounding mental health problems made little difference to the relationships between mental illness and QoL. Future studies might provide similar control analyses to further diffuse doubts about the influence of item overlap.

The naturalistic sample of this study has a number of advantages and disadvantages attached: while it is quite representative of real life patients, the distribution of internalising and externalising pathology turned out to be quite imbalanced and comorbid occurrence of internalising and externalising symptoms was high. Although participation rate was less than 50%, there did not seem to be a large selection bias in terms of age, gender, or pathology with affective disorders being the exception. Another limitation which should be emphasized is the inclusion of relatively severe cases which may have led to an overestimation of the association of QoL with internalising and externalising problems. Additionally, the sample size was relatively modest for the type and number of analyses conducted, hence caution is warranted in drawing conclusions from the analyses. Furthermore, the cross-sectional design prevents us from drawing conclusions regarding the direction of influence of factors on QoL.

To sum it up, psychiatric pathology is associated with the impairment of the child’s QoL. Thus, extending the outcome measures in psychotherapy research from mere pathology to measures of QoL might enhance sensitivity to the patient’s well-being and level of functioning. Especially internalising pathology was associated with impaired QoL compared with externalising pathology. In view of the predictors of QoL over and above pathology, one can conclude that with increasing age and with the persistence of psychiatric problems the consideration of QoL for the therapeutic process might gain in importance.

## Consent

Written informed consent was obtained from all participating patients (aged 11 years and older) as well as their parents.

## Competing interests

The authors declare that they have no competing interests.

## Authors’ contribution

KW carried out the data analyses and drafting of the manuscript. SWG and GR conceived of the study, participated in its design and coordination, and supervised the data analyses and drafting of the manuscript. JKD made significant contributions to the design of the study and critically revised the manuscript. All authors read and approved the final manuscript.
